# Population reduction by hunting helps control human–wildlife conflicts for a species that is a conservation success story

**DOI:** 10.1371/journal.pone.0237274

**Published:** 2020-08-11

**Authors:** David L. Garshelis, Karen V. Noyce, Véronique St-Louis

**Affiliations:** 1 Minnesota Department of Natural Resources, Grand Rapids, Minnesota, Unites States of America; 2 Minnesota Department of Natural Resources, Forest Lake, Minnesota, Unites States of America; Sichuan University, CHINA

## Abstract

Among the world’s large Carnivores, American black bears (*Ursus americanus*) are the foremost conservation success story. Populations have been expanding across North America because the species is adaptable and tolerant of living near people, and because management agencies in the U.S. and Canada controlled hunting and other human-sources of mortality. As a result, human–black bear conflicts (damage to property, general nuisance, threat to human safety) have dramatically increased in some areas, making it urgently important to develop and deploy a variety of mitigation tools. Previous studies claimed that legal hunting did not directly reduce conflicts, but they did not evaluate whether hunting controlled conflicts via management of population size. Here, we compared temporal patterns of phoned-in complaints about black bears (total ~63,500) in Minnesota, USA, over 4 decades to corresponding bear population estimates: both doubled during the first decade. We also quantified natural bear foods, and found that large year-to-year fluctuations affected numbers of complaints; however, since this variation is due largely to weather, this factor cannot be managed. Complaints fell sharply when the management agency (1) shifted more responsibility for preventing and mitigating conflicts to the public; and (2) increased hunting pressure to reduce the bear population. This population reduction was more extreme than intended, however, and after hunting pressure was curtailed, population regrowth was slower than anticipated; consequently both population size and complaints remained at relatively low levels statewide for 2 decades (although with local hotspots). These long-term data indicated that conflicts can be kept in tolerable bounds by managing population size through hunting; but due to the bluntness of this instrument and deficiencies and uncertainties in monitoring and manipulating populations, it is wiser to maintain a population at a level where conflicts are socially-acceptable than try to reduce it once it is well beyond that point.

## Introduction

The conservation and restoration of some wildlife species, especially Carnivores, has spurred mounting conflicts with humans in many places around the world [1–4]. Although human–wildlife conflicts (involving threats to humans or damage to property) have existed for millennia, they are now being reported in unprecedented numbers in some regions, partly as a result of species restoration occurring within the habitat constraints imposed by burgeoning human populations. Conflicts are especially amplified in the case of species that are able to live near humans and benefit from consuming human-related foods [5].

A prime example of a successfully recovered species that is now the subject of widespread conflicts with people is the American black bear (*Ursus americanus*). Conversion of forests and unregulated hunting and persecution led to extirpation of this species across large portions of its range by the early 1900s [6,7]. This trend was reversed as U.S. states and Canadian provinces gained control over unwanted and unsustainable killing, in part by “protecting” the species with big game status [8]. This meant that legal hunting could occur only with a license, and state and provincial agencies regulated the take with various restrictions, and policed other forms of take [9]. Conversion to a big game species was motivated not because the species was common enough to hunt, but rather to help build populations that could be sustainably hunted for recreation and meat, under what has come to be called the North American Model for Wildlife Conservation [8–11]. Today black bears are legally hunted (harvested) as a game species in all 12 Canadian provinces and territories where they exist and in 32 U.S. states. Six states opened bear hunting seasons since the early 2000s.

The recovery of American black bears in recent decades has been dramatic. By 1999, 60% of US states and Canadian provinces reported increasing populations, and most other jurisdictions appeared to be stable [12]. A more recent (2018–2019) tally indicates that two-thirds of states with resident black bears have increasing populations [13 Appendix V]. Eight U.S. states where this species was once completely extirpated now have viable populations, and all continental U.S. states have reported recent sightings of black bears [7]. Limiting human-caused mortality has been key in the successful recovery of this species. Equally important are the adaptations and tolerance of this species for living near people, as well as the growing tolerance of people toward black bears in their midst (i.e., their recognition that the species is rarely dangerous and their willingness to endure some potential inconvenience or damage). Indeed, one of the highest density populations of this species occurs in New Jersey [14], the state with the highest human density in the U.S.

The American black bear is now by far the most common wild large Carnivore on the planet [15]. A consequence, however, of the successful recovery of this species is increasing conflicts with people [16,17]. Black bears not only eat crops and sometimes kill livestock, but they also are attracted to household foods and garbage, pet food, birdseed, and camp food, and will destroy property in an attempt to obtain such food, especially in years when their natural forest foods are lacking. Black bears also occasionally injure or kill people. They often instill fear, especially in people who have had little direct experience with them; however, this species is more tolerated at close proximity than the more dangerous brown bear (or grizzly bear; *U*. *arctos* [18]), which is sympatric in parts of western North America.

A number of mitigation practices have proven to be successful for reducing human–black bear conflicts in specific situations [13,17,19–21], but studies have shown that despite intense public education, conflicts often remain due to imperfect or inappropriate use of these methods [22–25], or circumstances for which existing methods are not wholly satisfactory [26,27]. For this reason, management agencies may turn to lethal controls—either targeted killing of individual nuisance bears or hunting—as possible solutions, or at least as additional tools. The rationale for reducing human–bear conflicts through hunting is not that it will necessarily remove specific bears involved in conflicts, but rather that it can control population size, and it seems intuitive that fewer bears would create fewer conflicts. Studies of lethal killing of other depredating carnivores have often been inconclusive in terms of resulting conflicts in part because most did not evaluate effects on population size, and moreover, reduction of one carnivore sometimes caused replacement by others [28–30]. However, Herfindal [31] found that recreational hunting that intended and succeeded in reducing the population of Eurasian lynx (*Lynx lynx*) reduced depredations on domestic sheep.

The actual relationship between population size and human–bear conflicts remains equivocal because conflicts are driven by a host of other factors, such as availability of both natural foods and human-related foods, and proximity of bear habitat to humans. Sorting out the effects of these variables from the effects of bear numbers is difficult, especially if population size is not reliably measured over an adequate area for a sufficient period of time, or the population does not change sufficiently to cause a significant response in conflicts.

The bear literature regarding effects of hunting on conflicts has been muddled by studies with contradictory results. In New Jersey, black bear hunting was suspended for 33 years and the population grew and caused extensive conflicts; hunting was then reinstituted and halted multiple times over a period of recent years. Raithel et al. [32] found that total nuisance incidents in years following a harvest were less than in years following a closed hunting season; they also found that lethal control of nuisance bears contributed to reduced conflicts the next year. Conversely, Treves et al. [33] and Obbard et al. [34] concluded that human–black bear conflicts in Wisconsin and Ontario, respectively, were not correlated with previous harvests, and therefore surmised that hunting was ineffective at controlling conflicts. Likewise, Artelle et al. [35] found that increased killing of grizzly bears in British Columbia, Canada, did not reduce conflicts (here limited to attacks on people), and therefore argued that management agencies should reconsider hunting and other removals as a means of reducing conflicts. All three of these studies, though, dismissed the effects of population size. Treves et al. [33] analytically “sought to eliminate the confounding effect of the bear population estimate”, which they observed was positively correlated to complaints, so as to focus solely on whether raw harvest numbers affected subsequent complaints. Similarly, Artelle et al. [35] observed that “whereas areas with higher estimated densities of grizzly bears and humans experienced more conflict, annual hunting intensity had no measurable effect on subsequent conflict, suggesting attempted population reduction via hunting might not be effective in mitigating conflict”. It is unclear if they meant that failed attempts to reduce a population would have no effect, but successful population reductions might reduce conflicts.

Harvests can be increasing while the population is also increasing, leading to higher conflicts with higher harvests (seemingly suggesting a relationship opposite of expectation); but without harvests, the populations surely would have increased faster. A prime example today is the state of Florida, where the population of black bears is growing rapidly due to a lack of hunting, and conflicts surged to where they are now among the highest in the species’ range [13,36].

Here, using 4 decades of data, we compared temporal patterns in human–bear conflicts in the state of Minnesota to actual bear population estimates, both of which changed dramatically. Also, over the course of this prolonged period, natural food supplies for bears varied substantially year to year, and a major policy shift was instituted for dealing with conflict-related complaints. The extreme magnitude in each of these perturbations provided an uncontrolled experiment that led to a better understanding of the effect of these potential drivers. Our goal was to rigorously explore the effects of these factors on the number of complaints so as to provide improved guidance for managing harvests and conflicts in the future across the range of this species, as well as to highlight aspects of conflict management and monitoring that may be applicable to a wider suite of species.

## Materials and methods

### Study area

Black bears in Minnesota live primarily in the forested third of the state, mostly north of 46°N latitude. Dominant forest types are aspen (*Populus tremuloides*, *P*. *grandidentata*), northern hardwoods (including *Quercus* sp., *Acer* sp., *Pinus* sp., *Betula papyrifera*, *Abies balsamea*, *Fraxinus nigra*), and lowland conifer (*Picea mariana*, *Larix laricina*, *Thuja occidentalis*). Forested lands are owned privately (36%), by the state (24%), counties (16%), federal government (17%), or industries (paper, timber, 7%). The forest zone is embedded with numerous small towns and one medium-sized city (Duluth, population 86,000, which has resident bears and periodic influxes of bears [37]). Along the periphery of the forest zone is a forest-prairie transition, also inhabited by bears, that contains smaller patches of forest in a matrix of agriculture. Some bears in this region rely heavily on crops (mainly corn and sunflowers [38,39]), which has enabled the bear population in parts of this region to expand geographically into predominantly agricultural land, well beyond the edge of what is traditionally considered suitable (forest-dominated) habitat for black bears [40].

The current resident human population within the bear range totals about 900,000, at an average county-level density of 9 people/km^2^ (range 0.6–46.9). During the summer months, when bears are active, the human population is significantly increased by tourists and cabin-owners, especially along the state’s many lakeshores.

### Complaint data

Conflicts between people and bears were reported by the public to the Minnesota Department of Natural Resources (MDNR), typically by phone, and were handled either by Wildlife Managers or Conservation Officers. The MDNR (hereafter “we”) began tallying these reported conflicts (hereafter “complaints”) in 1981, and we have kept up this record-keeping since then. Reports from MDNR staff initially were submitted monthly on paper forms, subsequently as Word documents submitted by email, and finally entered directly (following each individual complaint) in an electronic, menu-driven database (beginning in 2017 and fully implemented in 2019). Complaints were registered only during April–October, as very few occurred in the other months, when bears were typically denning.

**Fig 1:**
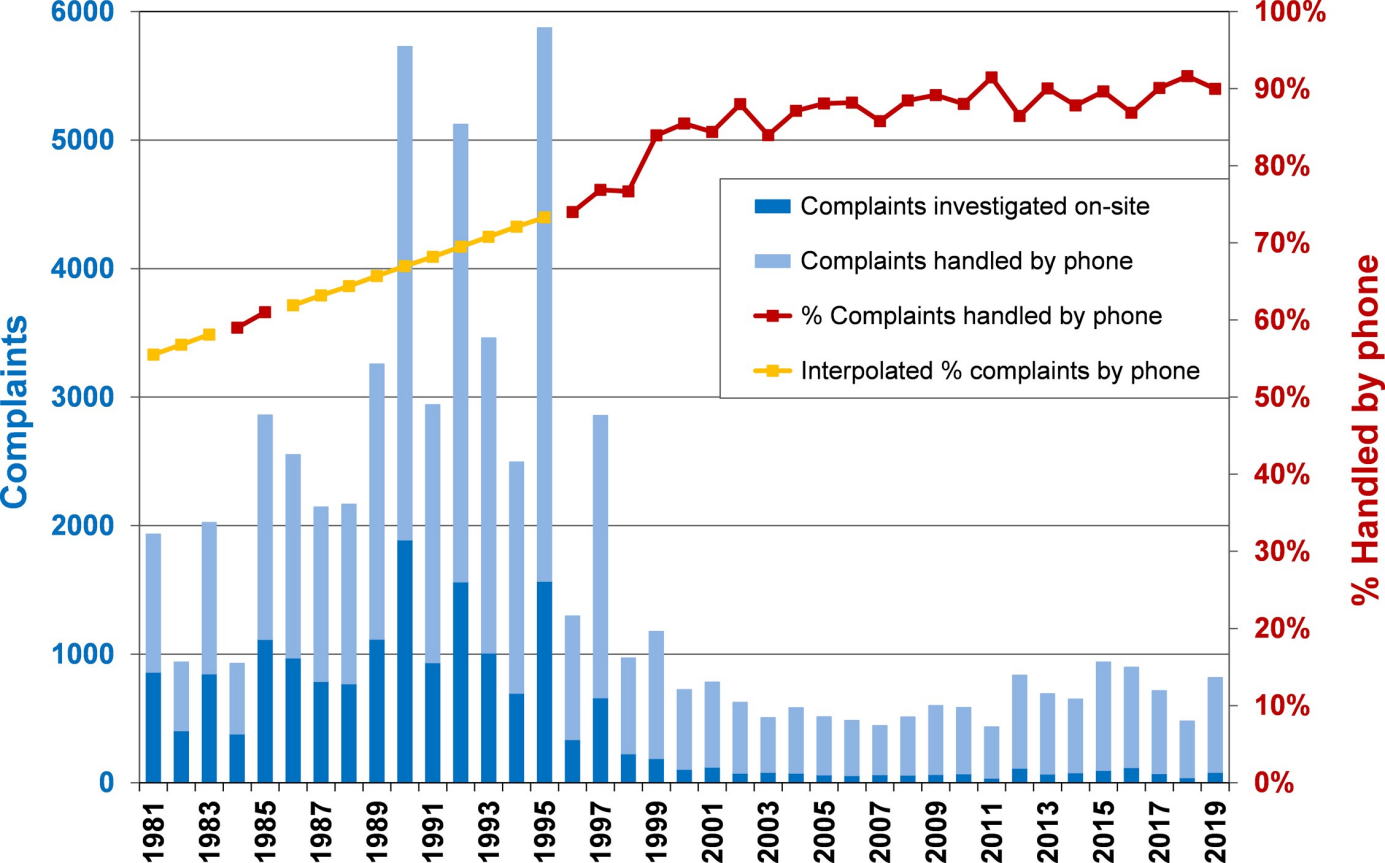
Complaints handled by phone versus on-site. Minnesota Department of Natural Resources personnel who received complaints about bears could investigate the situation on-site or handle it by phone. Over the years, an increasing percentage of complaints were handled by phone. A regression was used to estimate complaints handled by phone in years when only on-site visits were recorded.

MDNR personnel could either address a complaint by phone or visit the site to make an assessment and recommendation. During 1981–1995 (excepting 1984–85), MDNR staff only recorded complaints where an on-site visit was made. Subsequent years showed an increasing percentage through time of complaints handled only by phone (Fig 1). In order to estimate total complaints in the years where phone-handled complaints were not recorded, we regressed the percentage of complaints handled by phone during 1984–1985 (59–61%) to 1996–1998 (74–77%) and predicted the missing values (Fig 1).

Complaint records included the types of conflict involved, as perceived and reported by the complainant, in the following categories: threat to human safety, garbage, property damage, birdfeeder, campground, livestock, beehives, or crops. Sightings of bears were not included unless the complainant reported feeling threatened by the bear’s proximity or behavior. MDNR personnel responded to complaints and recorded one or more of the following actions: verbal advice provided, bear caught and translocated, bear killed or attempted to be killed and by whom.

During 1998–2000, we transitioned to a new policy, reducing site visits and eliminating translocations of bears in an effort to shift the responsibility for conflict resolution from MDNR to homeowners and landowners. We focused on providing verbal advice about removing or securing attractants. However, Minnesota state law allows people to kill bears, without seeking advance permission, if the bear is doing damage to their property or perceived to be threatening to do damage or harm to people. Individuals are required to report killing of a bear within 48 hours, and are not permitted to keep the carcass. Most killing of so-called nuisance bears is done by private individuals, although some are killed by MDNR Conservation Officers, local police, or hunters (in a program allowing them to hunt before or after the bear hunting season, mainly to target bears in crop fields).

### Hunting and bear population data

Minnesota’s bear hunting season spans 6–7 weeks from September 1 to mid-October. Bears can be hunted only with a bear hunting license. Since 1982 bear hunting licenses have been allocated by quotas for specific Bear Management Units within the primary bear range. Since 1987, license sales have been unrestricted along the periphery of the bear range. Hunters can shoot one bear on a license, and baiting is allowed and used by ~90% of hunters (dogs are not allowed). Other than baiting, which occurs during and 2 weeks prior to the hunting season, there is no widespread supplemental feeding.

Quotas on license sales are adjusted each year in accordance with population goals and population trajectories (described below). Quotas for specific Bear Management Units were increased when complaints showed an increasing trend in an effort to target areas where local populations were perceived to be too high. However, local bear population densities were, for the most part, unknown.

Bear hunters were required to register their bear, identify the sex, and submit a premolar tooth for age estimation, which is done at a MDNR lab. Registration compliance was believed to exceed 95%. Compliance in tooth submission varied from 65–90%. In some years we made an effort to obtain samples from hunters who did not initially comply, by reminding them that the tooth submission was mandatory. The age distribution of these follow-up samples did not differ from the initial samples, indicating that although we did not have a tooth from every harvested bear, the sample we had was not biased with respect to age. However, we found, from the harvest of known-sex radio-collared bears, that hunters misreported the sex of 11% of females (but rarely mistook the sex of males), so we corrected sex-specific harvest totals accordingly [41].

We used the sex-specific harvest data and sex-specific age distribution of harvested bears in a Downing [42] population reconstruction model to generate a trajectory of the bear population. We combined all ages 3 years and above, as this allows the model to produce estimates up to 2 years prior to the most recent harvest (e.g., up to pre-hunt 2017 for data through the 2019 harvest), and collapsing to this age still shows an unbiased trend based on computer simulations [43]. The Downing model estimates the number of living bears each year that eventually die due to hunting, but does not include bears that ultimately die due to other causes. From a sample of 387 radio-collared bears monitored until their death (1981–2019) across four study sites in Minnesota, 76% died from hunting [44], but since most of our study bears lived in an area where they were exposed to higher-than-average hunting pressure, we estimated that other forms of mortality were higher for the statewide population. We added an estimate of non-hunting mortality (35% of total mortality) to our population estimates; however, since this adjustment was the same for each year, it did not affect the shape of the curve (year-to-year magnitude of change), which is what we compared to the complaint data. The humped shape of the Downing population curve was supported by (a) four statewide population mark–recapture estimates [45] at 5–6 year intervals (1991, 1997, 2002, 2008), (b) results of an integrated population model that, besides age-at-harvest data, also included hunting effort, natural food abundance, and the statewide population estimates [41], (c) results of a Bayesian population model that included age-at-harvest data along with reproductive and survival estimates [46] that we derived from our long-term telemetry studies [44], and (d) a series of local mark–recapture population estimates on a study site near the center of the bear range [47–49]. The Downing model also provided estimates of the sex-age composition of the living population.

### Bear food data

It is well established that conflicts between American black bears and people are exacerbated by periods of scarcity of natural foods [3,34,37,50–52]. We thus obtained an index of food abundance each year across the Minnesota bear range. During April–June, bears in Minnesota consume young green vegetation and insects [51], but we had no means of measuring availability of these spring foods; moreover, though their abundance certainly varied to some degree across years, these foods were always plentiful across the landscape throughout the spring season, but may have varied in quality. During July–October bears consumed a variety of wild fruits and nuts, which, in contrast, varied visibly and markedly in abundance year to year. Each year, MDNR wildlife managers and foresters made categorical subjective assessments, within their work area, of the abundance of 14 key fruit-producing species (or groups of similar species), including herbaceous plants, shrubs, and trees; they also assigned a separate score for the fruit productivity of each. Both ratings were on a 0–4 scale (absent to abundant), and were multiplied together to achieve a food abundance rating for each species [53]. The species-specific food ratings were averaged among survey participants and then all species were summed to obtain yearly numerical food ratings ($\bar{x}$ = 62.8, range = 42.7–87.6), which we used here in a quantitative analysis. We also examined the distribution of these yearly ratings and divided them into three broad categories (poor, normal, abundant), used here in a qualitative overview of factors potentially influencing conflicts.

### Model testing

We used multiple linear regression and Akaike Information Criteria to compare the relative strength of candidate models in predicting the number of complaints received each year (COMPLAINTS). We explored models containing the following predictor variables: (1) estimated size of the bear population (POP); (2) yearly numerical rating of bear foods (FOOD); (3) the number of bears removed from the population the previous year through hunter harvest or killing in conflict situations (PREVKILL); and (4) the MDNR nuisance bear policy in effect (POLICY: old or new, pre- and post-1998; categorical variable). In the first set of candidate models tested (n = 11; see S1 Table), spanning 1982–2017, we also included a fifth variable (POPLEVEL) that stratified the population as either <15,000 or >15,000 bears (categorical). We added this threshold variable because the data suggested that the magnitude of year-to-year variability in complaints might be more extreme at high bear densities and muted at lower densities.

We tested a second set of candidate models (n = 16; see S2 Table) using the four initial variables (POP, FOOD, PREVKILL, and POLICY), and added a variable for the estimated number of ≥6-year-old females in the population (POPF6). Inclusion of this parameter limited the data set to 1982–2014 (due to the restrictions of the Downing reconstruction). We explored this model because we knew that the female age structure changed considerably over the duration of this study. Our previous analysis indicated that whereas 72% of nuisance bears that were captured or killed were males, among females, older animals were disproportionately represented compared to their occurrence in the living population [37]. Likewise, a study in western U.S. found that as female black bears age, they become more attracted to human food sources [54].

Given this is a time series, where events one year could affect the next, we examined plots of the autocorrelation function (ACF) of the regression residuals at various time lags for all top models. We also examined the top models for uninformative parameters [55,56]. All statistical analyses were performed in the statistical program R [57]. AIC(c)-based model selection was done using the MuMIn library [58] and ACF plots were made using the ggplot2 library [59].

## Results

### Trends in conflicts

**Fig 2:**
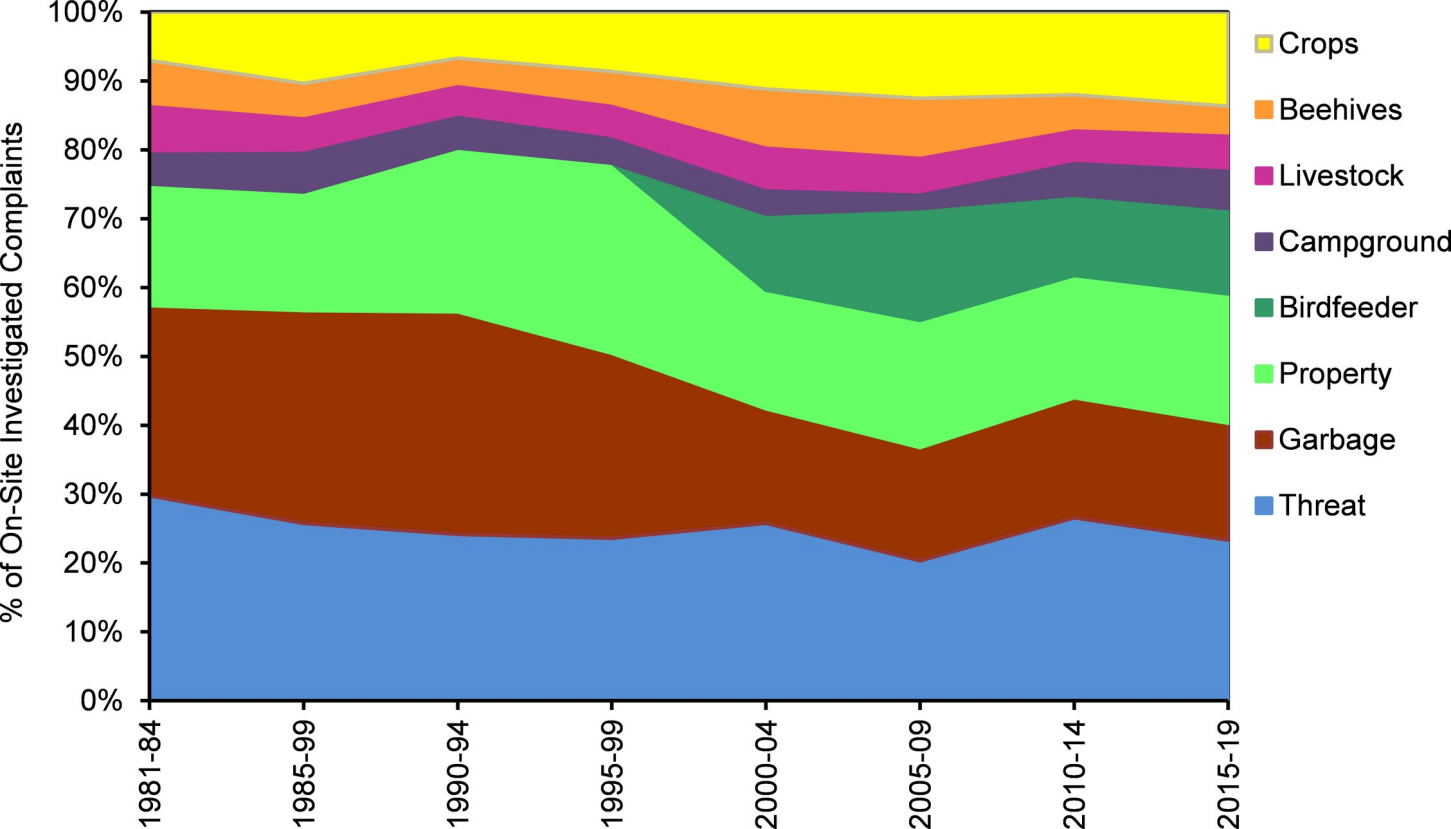
Changes in the percentage of different types of human–bear conflicts investigated in Minnesota, 1981–2019. Complaints concerning bears in garbage diminished while birdfeeder complaints increased.

**Fig 3:**
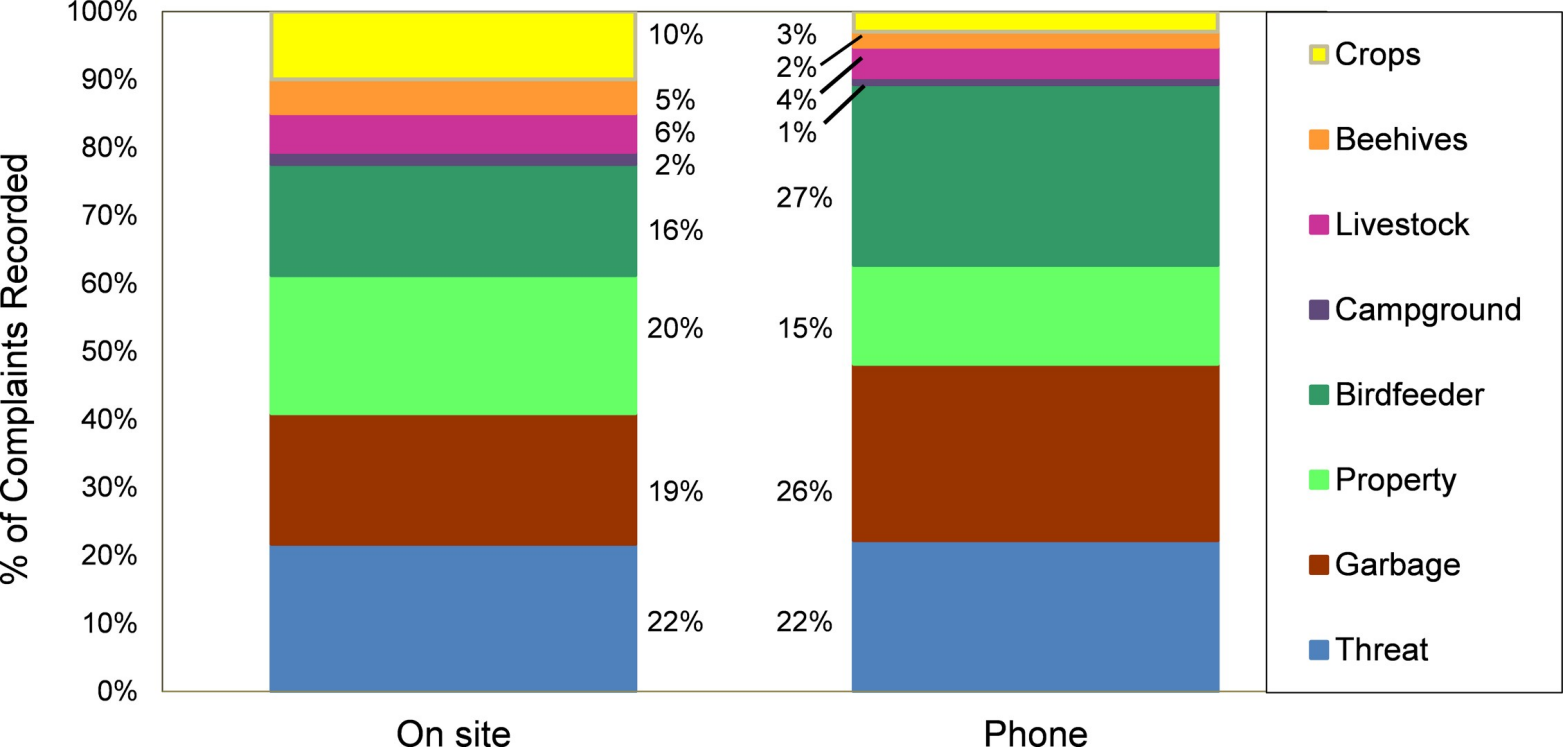
Types of complaints handled by phone versus on-site. Recent data (2016–2019), shown here, indicate that complaints involving bears damaging property, beehives, or crops prompted disproportionately more on-site visits by the Minnesota Department of Natural Resources, whereas advice about deterring nuisance activity (garbage, birdfeeders) was often provided by phone.

The MDNR received ~63,500 bear-related complaints during 1981–2019. During the 1990s, a noticeable decline occurred in the percent of complaints involving garbage, as educational efforts were directed at better securing this bear attractant; however, this food source was replaced during the 2000s by birdfeeders, as bird feeding became an increasingly popular summer recreation in northern Minnesota (Fig 2). The relative contribution of other types of complaints remained fairly stable through time, led by threat to human safety, being of paramount concern to about a quarter of complainants. MDNR personnel were more likely to visit sites when damage occurred to crops, beehives, or property versus complaints about bears that were considered a nuisance (e.g., garbage, birdfeeders) or a safety threat (Fig 3).

Before the MDNR policy change in 1998–2000, an average of 6% of complaints led to a bear being translocated (range 50 to >350 bears/year; $\bar{x}=168$), and 7% resulted in a bear being killed (range 30 to >360 bears/year; $\bar{x}$ = 173). From the year 2000 onwards, translocations were eliminated except in a few unusual circumstances, and bears were killed in 4% of complaints (range 9–53 bears/yr; $\bar{x}$ = 26). In the last 10 years of the data (2010–2019), an average of only 11% of complaints were visited on site by MDNR staff (Fig 1).

**Fig 4:**
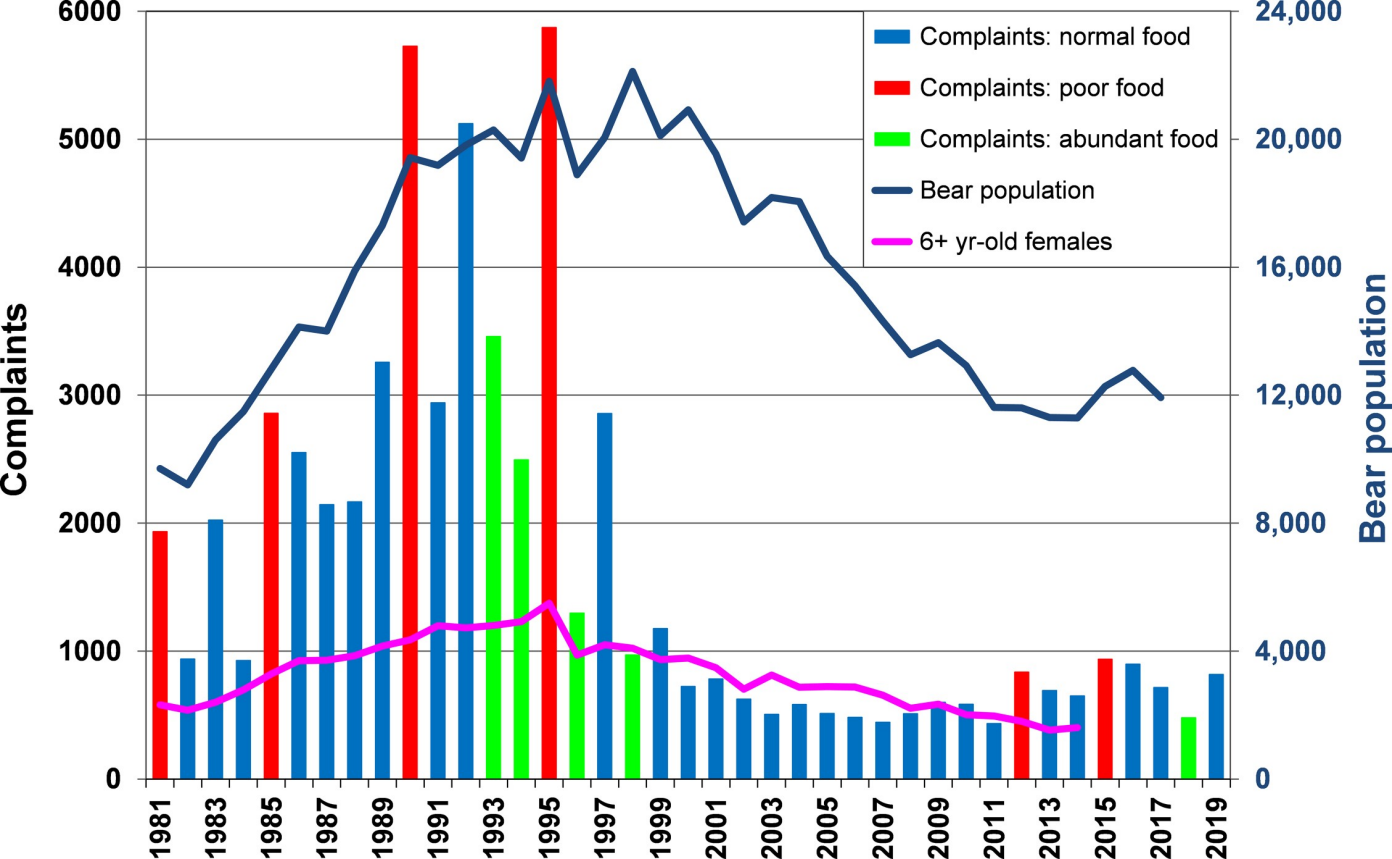
Comparison of total bear complaints, population size, and yearly rating of natural foods. Bear complaints rose sharply as the population of bears rose (population estimates not available for most recent 2 years). Complaints were especially high in years when natural summer and fall foods for bears were sparse, and were low when foods were abundant. A sharp decline in complaints occurred during 1998–2000 when the MNDNR phased-in a policy against translocating bears and greatly reduced on-site visits (Fig 1). Reduced complaints also corresponded with fewer prime-age females in the population.

A generally increasing trend in complaints was evident through the 1980s, peaking in the mid-1990s (exceeding 5,000 in 3 years). This matched the trend in the bear population, which doubled in 10 years (Fig 4). Four poor food years occurred during this increase phase, and complaints during each of these years exceeded the number of complaints during adjacent normal food years, and were more than twice that of adjacent years with abundant natural foods (Fig 4). The number of complaints in the four poor food years, spaced at 4–5 intervals (1981, 1985, 1990, 1995) increased commensurate with the steep population increase.

Complaints dropped off quickly from the late 1990s to the early 2000s, coinciding with three events: (1) two years with unusually abundant foods (1996, which was the best food year in all regions of the state, and 1998), (2) the MDNR policy change intended to shift responsibility for conflict mitigation to the public (thereby discouraging complaint calls), and (3) a decline in the bear population. This population decline, which led to a near halving of the population in 15 years, lagged a few years behind the initial drop in complaints.

**Fig 5:**
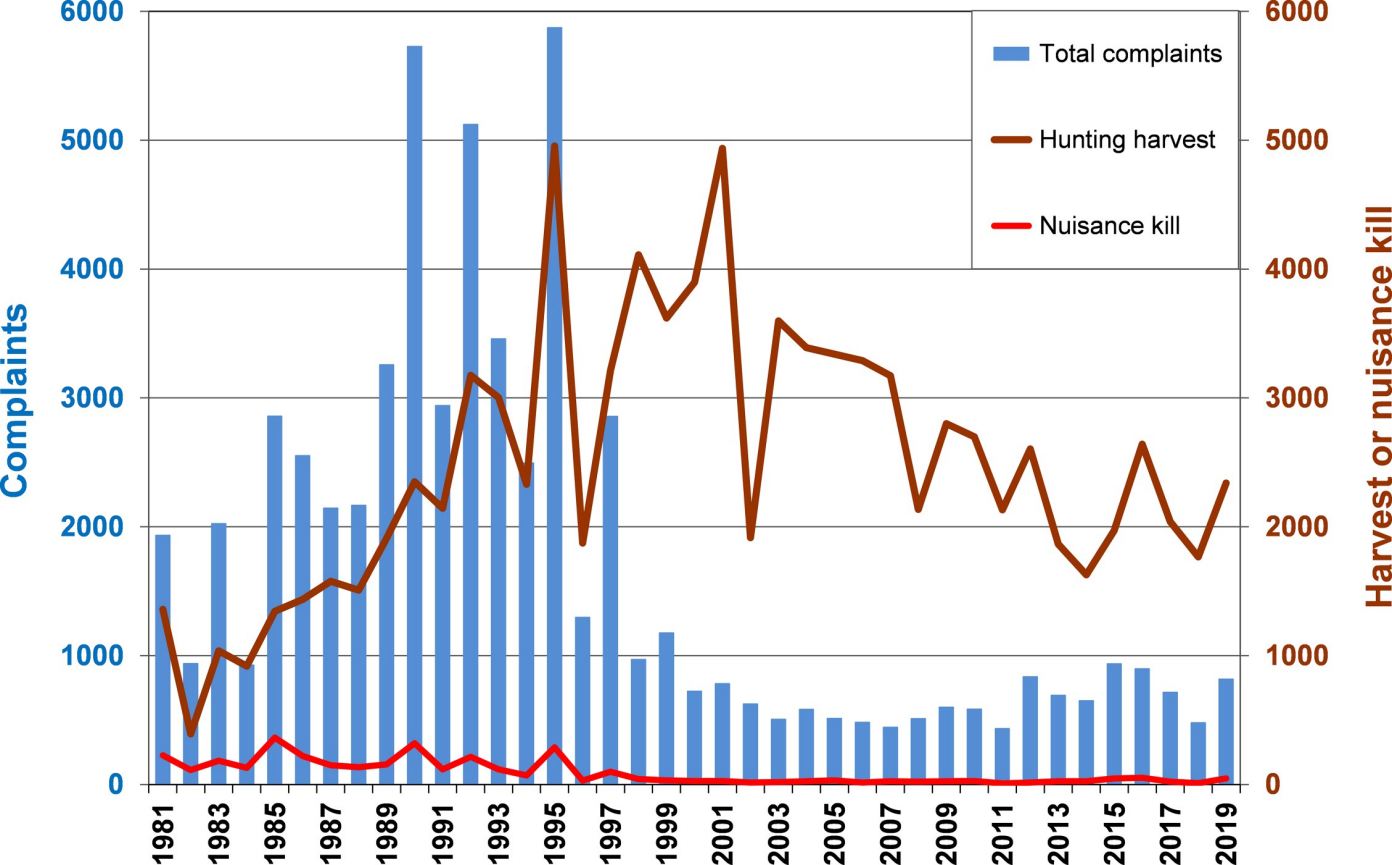
Bear complaints not directly related to harvest. No relationship was evident between harvests and bear complaints because increasing harvests during the 1980s through early-1990s did not cause a population decline. However, in 1995 an especially high harvest combined with a high kill of nuisance bears probably helped to reduce human-bear conflicts for a few years.

A fourth likely factor in the decline in conflicts was the effects of the excessive bear killing that occurred in 1995, the peak complaint year. Nearly 300 bears were reported killed that year in conflict situations spurred by the paucity of natural foods, combined with a record high fall harvest of nearly 5,000 bears, also due to poor foods that prompted greater attraction to hunters’ baits (Fig 5). In total about one-quarter of the state’s bear population was removed that year. Moreover, prime-age 6+-year-old females made up an unusually large portion of the 1995 harvest (26% of total harvest, 45% of female harvest)—higher than any year before ($\bar{x}$ = 17% of total harvest) or since ($\bar{x}$ = 13%). Whereas the bear population as a whole did not start its steep decline until 2001, decline in the number of prime-age females began immediately after 1995 and continued steadily through the remainder of the study period (Fig 4). Males did not show a shift in age structure.

With the exception of a few key years like 1995, there was no evident relationship between harvest in one year and complaints in ensuing years (Fig 5). In fact, harvest increased during the 1980s to mid-1990s while conflicts also generally rose, because the harvests did not cause a population decline. Sizeable fluctuations in harvests in the most recent 2 decades are not reflected in numbers of complaints, which have remained relatively low and stable. Two poor food years (2012, 2015) yielded a small bump in complaints, and one abundant food year (2018) produced a dip (Fig 4), but the magnitude and year-to-year variation in complaints was minimal compared to the early 1990s, when the population was at its maximum.

### Quantitative identification of conflict predictors

The top explanatory models (ΔAIC(c) ≤ 4) explaining the number of complaints received by MDNR personnel each year all included a measure of population size (POP or POPF6), food availability, and MDNR policy (Table 1). We tested for, and found no significant temporal autocorrelation (95% confidence intervals around ACF estimates) in the residuals of these top models at a 1-year time lag. In model set 1 (1982–2017), adding the binary variable POPLEVEL (threshold of 15,000 bears in population) improved the model fit and explained 76% (adjusted model R^2^) of the variability in year-to-year conflicts. In model set 2 (1982–2014), the number of females ≥6 years old (POPF6) was as good or better a predictor of complaints as the total population size (POP); models that included both of these variables did not fit the data as well. Variance inflation factor (VIF) values suggested high multicollinearity when both of these variables are included in the same model (VIF = 14.5 and 22.2 for POP and POPF6, respectively; VIF < 3 when only one of the two is included). In both model sets, including the previous year’s bear kill (PREVKILL) raised AIC(c) by ~2 and did not improve model fit, suggesting that it was likely an uninformative parameter, thus providing further evidence that complaints are generally not directly driven by previous harvest, but by how that harvest affects the population size.

**Table 1:** Best-ranked of candidate models (ΔAIC(c) ≤ 4) explaining number of complaints about human–bear conflicts in Minnesota.

Candidate models	*k*	AIC(c)	ΔAIC(c)	*w*_i_	Cum *w*	adj R^2^
**MODEL SET 1: 1982–2017**						
POP + FOOD + POLICY + POPLEVEL	6	587.5	0	0.54	0.54	0.76
POP + FOOD + POLICY	5	589.5	2.0	0.20	0.74	0.73
POP + FOOD + POLICY + POPLEVEL + PREVKILL	7	590.3	2.8	0.13	0.87	0.75
POP + FOOD + POLICY + PREVKILL	6	591.1	3.6	0.09	0.96	0.73
**MODEL SET 2: 1982–2014**						
POPF6 + FOOD + POLICY	5	541.7	0	0.33	0.33	0.74
POP + FOOD + POLICY	5	542.3	0.7	0.24	0.56	0.74
POPF6 + FOOD + POLICY + PREVKILL	6	544.3	2.7	0.09	0.65	0.74
POP + POPF6 + FOOD + POLICY	6	544.5	2.9	0.08	0.73	0.74
POP + FOOD + POLICY + PREVKILL	6	544.6	2.9	0.08	0.80	0.74

All top models included at least one variable related to bear population size (POP = total population; POPLEVEL = bear population >15,000 or <15,000 [included in model set 1 only]; POPF6 = population of 6+-year-old females [included in model set 2 only]); availability of wild bear foods during summer and fall (FOOD); and whether the MDNR revised nuisance bear policy was in effect (POLICY, pre- or post-1998). The total number of bears killed the previous year (PREVKILL = total human-caused mortality due to hunting and nuisance) appeared in some top models but did not add explanatory power.

## Discussion

A controversy over whether hunting helps to alleviate human–bear conflicts has permeated the literature and confused management agencies and the public in North America. Hristienko and McDonald [16] argued that escalating conflicts with American black bears were largely related to expanding bear populations, so it logically follows that some population control would help to limit conflicts. Two later studies [33,34], though, claimed to have refuted this paradigm with empirical data showing that harvest did not affect complaints. Both of these also cited our earlier study in Minnesota [37] as originally showing that harvests did not limit conflicts; in truth, our earlier paper simply presented a graph showing simultaneously rising hunter harvests and nuisance kills, which was the impetus for more regulation (leading to establishment of hunting license quotas in 1982). Misinformation and flawed study designs have heretofore obfuscated an understanding of the role of harvest in conflict management. Our aim here was to re-examine the evidence, using a longer dataset with more variation.

Three primary factors stood out as influencing the number of complaints concerning human–bear conflicts: bear population size, natural food conditions, and a policy shift designed to influence the public’s perception about what they could do on their own versus help that they could expect to receive from the government to deal with bears they considered a problem. Previous studies that discounted population size as a driving factor either had no measure of population size (and incorrectly used harvest as a surrogate), or examined a situation where population size was not diminished by harvest. We had a rather unique situation in which the statewide population of bears doubled (from about 10,000 to 20,000) in one decade, during which time complaints more than doubled (from about 1,500 to >4,000).

During this sharp rise in complaints, the MDNR began implementing some management strategies to mitigate the conflicts, which were a growing annoyance to the public and a burden on agency personnel. The primary strategy was to lower the population through increased harvest (by increasing the quotas on hunting licenses). Harvests increased during the 1980s through the late 1990s (Fig 5). This resulted in a leveling of population growth by the beginning of the 1990s (Fig 4), after which the population remained at a high plateau into the early 2000s, and then fell rapidly with the continuation of relatively high harvests through about 2007. These harvests may themselves have removed a large proportion of bears that were prone to be a nuisance: 90% of harvested bears were taken over bait, so it seems probable that those most attracted to bait were bears that were accustomed to consuming human-related foods in other situations. In another Minnesota study we observed that bears that consumed research baits in summer were more likely than other bears to be shot by hunters that fall [45,60].

Heavy harvests also shifted the age structure, particularly for females. We found that a decline in prime, reproductive-age females preceded that of the population as a whole. Previously we observed that although harvests were almost always male-dominated, especially high harvests of old females occurred in years with poor foods during the hunting season [53]. Adult females, subject to the extra nutritional demands of cub rearing, may exhibit greater sensitivity to poor food conditions than other bears, particularly when high bear densities may cause food competition. Additionally, others have shown that as female black bears age and gain experience, they become more likely to learn and become attracted to the benefits of human-related sources of food [54]. Thus, while it was not the intent of the MDNR to reduce prime reproductive females, and whereas this was likely part of the reason for the prolonged population decline, it may have acted synergistically with the population reduction to reduce conflicts.

A rapid decline in complaints also coincided with a significant change in MDNR policy. MDNR public awareness messages about securing garbage apparently helped reduce this type of complaint through the 1990s (Fig 2). But it was the institution of a new MDNR policy, initiated in 1998 and fully implemented in 2000, that helped curtail overall complaints. The new policy was aimed at shifting the responsibility for conflict mitigation to the public: MDNR staff reduced time-consuming site visits and eliminated translocations of nuisance bears. The vast majority of people who did call indicated that they desired a non-lethal solution to the conflict. It is possible that through time, more and more northern Minnesotans learned how to avoid conflicts with bears. It is also possible that people’s attitudes, tolerance, and inclination to call for assistance evolved over time with their gained experiences [61].

Variations in natural food conditions also affected conflicts by altering the attraction of bears to human foods, and possibly affecting their behavior for several years. Major food failures at ~5 year intervals, from 1981–1995, may have forced bears to become more reliant on human foods, a behavior that some bears continued to employ even in normal food years, and likely passed to their offspring [62,63]. The period of rapid decline in conflicts included two abundant food years followed by an extended period of normal food conditions, enabling two generations of bears to find sufficient sustenance without supplementation of human-related foods.

Although we could identify the main predictors of complaints, it was not possible, nor the intent here, to determine which had the greatest impacts, as multiple factors changed at about the same time. Additionally, some factors were related to bear behavior, and some to human behavior, and none could be measured with precision. Food availability, in particular, was difficult to quantify. We could not quantify spring foods (before ripening of berries), and our assessment of summer-fall foods relied on a subjective assessment of prevalence and productivity of the main fruit-producing species, multiplied together. All of these fruit-producing species were then equally weighted, even though some appeared to be more important to bears than others [51]. Moreover, although fruit production was rated in categories on a linear scale, we found by counting fruits per unit area that our scale was not linear, but that each category represented 2–4 x the fruit biomass of the next lower category; hence, the difference between fruit biomass for a rating of 4 versus 3 was much greater than for 3 versus 2 [64]. Also, we did not account for sizeable spatial differences in food production; in some years, complaints spiked in just one region of the state where some key foods produced poorly. Finally, while it is useful to understand the effects of foods on conflicts, natural foods are not easily managed, as abundance of the specific food-producing plants varies with soils and forest management, and production of fruits and nuts varies enormously with weather as well as some inherent reproductive properties of each species. Thus, monitoring foods is more useful in understanding rather than controlling conflicts (e.g., not overreacting during a poor food year with many complaints).

It is important to add that our record of complaints relies on a chain of events: a bear seeking food near people, a person considering the bear a nuisance and being motivated to call either for advice or to complain, and that complaint being registered. Sometimes the public contacted local police or MDNR personnel who did not regularly deal with bears, and some of our staff did not record all complaints received. In some cases MDNR staff indicated that a rash of complaints over a short time span caused some recording to be missed. Clearly, our record of complaints is a very imperfect measure of actual conflicts.

**Fig 6:**
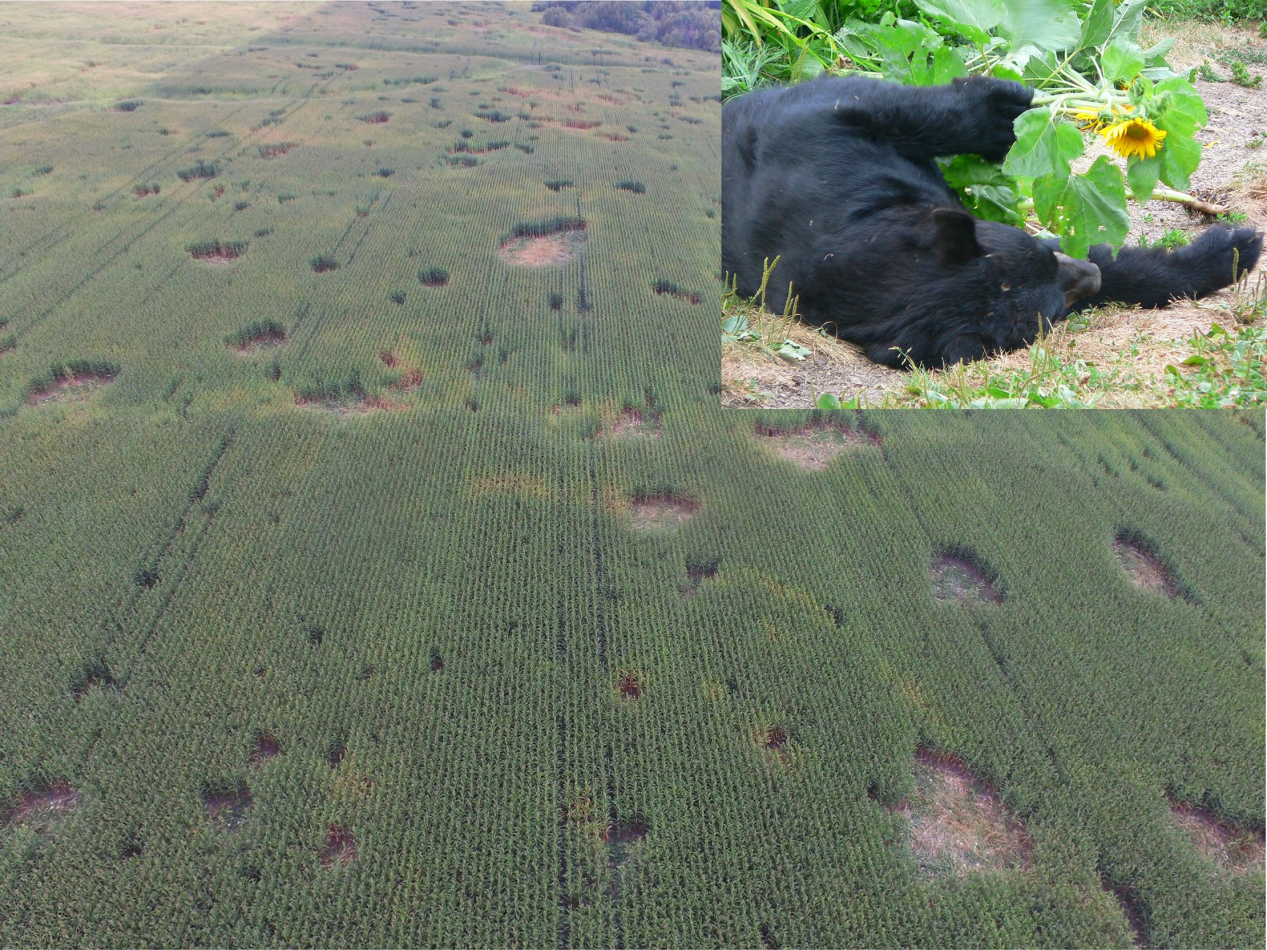
Bear damage to large crop fields. Damage to corn or sunflowers is difficult for individual farmers to prevent or even detect (here viewed from a drone). This situation is most apt to require lethal control. (**Inset**) Bears in crops are difficult to see, and our research indicates that they feel comfortable feeding in such situations due to the heavy cover [39]. Photos: MDNR and D. Garshelis, respectively.

Finally, it is useful to recognize that hunting not only affects populations, but also public perception of the agency’s response, and hence the public’s tolerance and propensity to register a complaint. For example, Ontario, Canada, witnessed higher complaints about bears after their spring hunting season was closed; this may have been due, in part, to the public anticipating more conflicts following this highly publicized and controversial action, and therefore being more apt to report conflicts [65,66]. Conversely, increasing hunter pressure in an area with increasing conflicts, especially conflicts regarding crops (Fig 6) which are difficult for individuals to mitigate, is likely to be viewed positively by the affected public, and could reduce complaints even if damage is not significantly reduced [26].

We recognize that population control may not be a socially-acceptable way to reduce conflicts in all areas. Since black bear conflicts tend to arise from human-related attractants or activities, an argument can be made that modifying human behavior is the ethically appropriate solution, especially if the harm caused by bears is relatively small (e.g., mainly a nuisance) [67]. A logical counter-argument, though, is that conflicts are caused by black bears exploiting a wide range of human-related food sources and environments, and this has contributed to their population growth [38,40,52,54,68–71] so controlling population size or expansion may be warranted when other mitigation efforts are inadequate. Also, there is a growing body of evidence suggesting that reduced hunting of black bears and brown bears in both North America and Europe may have relaxed artificial selection, enabling more bold individuals to survive, leading to more conflicts including attacks [72]. Feeding bears to draw them away from potential conflict situations is sometimes advocated, but this is also not without ethical concerns in that it alters natural bear behavior, artificially increases their nutrition and reproduction, and may grow a population above the environmental carrying capacity [73]. Our purpose here was not to advocate population control or the policy that we instituted for dealing with complaints, but just to show that these efforts worked to reduce complaints as well as the direct killing of nuisance bears—outcomes that were not assured when we started these management actions. Each jurisdiction with an expanding bear population must determine, based on their local situation, which approach or combination of approaches is best for mitigating rising conflicts that stem, in part, from rising bear populations. The practical and ethical dilemmas of how to prevent and mitigate conflicts resulting from successful species management is not just pertinent for American black bears, but also for a number of species where conservation efforts have restored populations to the extent that conflicts are difficult to control with local mitigation efforts.

## Conclusions

We showed empirically, for the first time, that complaints related to human–bear conflicts increased on a landscape level with an increasing bear population; in fact, conflicts more than doubled as the bear population doubled. Remarkably, this relationship was evident despite highly-varying food conditions, and despite wide variations in local bear densities and local situations (e.g., areas where many people fed birds while bears were active, areas where MDNR staff were less willing to provide advice or register complaints). We made no attempt to filter or subset the data to adjust for any local factors.

Conflicts were appreciably reduced when the population drastically declined due to hunting; this occurred in concert with a policy change but little additional effort at public education and virtually no government assistance with mitigation, other than providing verbal advice. To be clear, while the goal at the time was to reduce the statewide population due to the burgeoning complaints, the halving of the population was much more rapid and severe than anticipated or desired, and became difficult to arrest. The liberal hunting quotas through the 1980s and 1990s were in reaction to the very rapid population increase and corresponding unmanageable numbers of complaints, which showed no signs of abating, based on our best population estimates and models at the time. Indeed, the overshoot in hunting harvests is one of the lessons learned from this inadvertent experiment: harvest can be a tool to reduce conflicts via population reduction, but it can be challenging to manage harvests due to inherent difficulties in accurately tracking population size and composition in real time, and not being able to control or predict food conditions, which can significantly affect hunting success [53]. It also requires a major population change to witness effects, which is likely to be unwarranted on a large scale. Given the many uncertainties in population management and bear biology, agencies in North America have (with some exceptions) generally opted to manage black bear harvests conservatively, but increasing conflicts may pressure agencies to be more liberal [16], and with that, there is a growing need for more rigorous monitoring as well as better understanding of population dynamics. As an example, recovery of the bear population in Minnesota has been unexpectedly slow, possibly due to an extended perturbation of the age structure caused by the heavy hunting.

With sizeable reductions in bear hunting license quotas, the Minnesota population appears to be making a slow comeback (Fig 4). Accordingly, some local areas are now once again experiencing heightened conflicts. Although there are certainly a number of factors involved (e.g., unsecured attractants, bears appearing in areas where local people have not seen them before), the increase in complaints and sightings have generated a strong sense by both the public and local MDNR Wildlife Managers that a population increase is largely the cause, and once again there have been calls for a reduction. This highlights an important warning on the interpretation of this study: whereas it is logical that bear population size is a driver in the magnitude of conflicts, an attempt to manage population size through hunting in a local conflict hotspot is likely to be difficult and ineffective if the attractants remain there (making the area an attractive population sink). Moreover, short of establishing population studies within each local area where harvest is increased in response to conflicts, there is no effective means of gauging how much an increased harvest affected population size and trend. Across the range of bears (not just American black bears), it is common that agencies managing harvests lack precise population monitoring tools [12,74,75], and without these, intensive harvests directed at relieving conflicts could be a risky option.

A recommendation stemming from experiences in Minnesota is to mitigate local conflicts through targeted measures aimed at changing human behavior, reducing availability of attractants, and increasing tolerance of people, while at the same time managing and monitoring the population on a larger scale at a socially-acceptable level. We suggest that for American black bears, in states and provinces where they are prevalent, hunting can serve a role in setting broad bounds on the extent of conflict. For example, in Minnesota, a population exceeding 15,000 bears seems to represent a threshold for higher numbers of complaints (Table 1). It is fitting that regulated hunting, which helped rescue this species by shifting the killing of bears as varmints to harvesting them as a valued resource [8,9], should now be looked upon for tempering the new challenge. It is a blunt instrument, but an instrument nonetheless that can be useful if combined with other courses of action, innovative ideas, and partnerships between government agencies and other concerned entities [76,77].

A final important caveat stemming from the Minnesota experience concerns the essential need for reliable monitoring of any population that is managed through hunting to achieve a desired objective, such as limiting conflicts. There has been a long history of Carnivore population reductions aimed at controlling conflicts, much of it mired with mistakes, yet extending to the modern era [1,78]. Despite the best of intentions, many agencies have been unable to achieve population management objectives due to inadequate monitoring methods and inherent uncertainties [79]. This was our situation in Minnesota, where, even with 4 decades of research and monitoring efforts, and having control over the number of hunters, the hunted bear population dropped more quickly than desired and the recovery has been slower than expected. This underscores the need for caution in using population manipulation to control conflicts. Learning this lesson in Minnesota, we now aim to maintain a population that is more resilient to occasional over-harvests from high hunting success in poor food years—as a consequence, bear numbers in some local areas may need to be higher than desired in terms of conflict management.

## Supporting information

S1 Data (XLSX)

S1 Table Candidate models explaining number of complaints about human–bear conflicts in Minnesota, 1982–2017 (including variable POPLEVEL). (DOCX)

S2 Table Candidate models explaining number of complaints about human–bear conflicts in Minnesota, 1982–2014 (including variable POPF6). (DOCX)
