# Supplementary material for: Population reduction by hunting helps control human–wildlife conflicts for a species that is a conservation success story
Source: PLoS One. 2020 Aug 11;15(8):e0237274. doi: 10.1371/journal.pone.0237274 (PMC7418986; doi:10.1371/journal.pone.0237274)
Supplement: S1 Table — (DOCX) [file pone.0237274.s002.docx]

**S1 Table.** **Candidate models explaining number of complaints about human–bear conflicts in Minnesota, 1982–2017 (including variable POPLEVEL). Models with ΔAIC(c) ≤ 4 are bolded.**

| Model | Intcept | Coefficients | | | | | k | logLik | AIC(c) | ΔAIC(c) | w | Cum w | adj.R^2^ |
| --- | --- | --- | --- | --- | --- | --- | --- | --- | --- | --- | --- | --- | --- |
|  |  | pop | food | policy | pop  level | prev  kill |  |  |  |  |  |  |  |
| **Pop + Food + Policy + Poplevel** | 1847.1 | 0.3 | -51.9 | -1902.1 | -1218.7 | NA | 6 | -286.29 | 587.48 | **0.00** | 0.54 | 0.54 | 0.76 |
| **Pop + Food + Policy** | 3342.0 | 0.2 | -48.6 | -1887.8 | NA | NA | 5 | -288.75 | 589.51 | **2.03** | 0.20 | 0.74 | 0.73 |
| **Pop + Food + Policy + Poplevel+ Prevkill** | 1617.7 | 0.3 | -46.7 | -1802.8 | -1126.3 | -0.1 | 7 | -286.13 | 590.27 | **2.79** | 0.13 | 0.87 | 0.75 |
| **Pop + Food + Policy + Prevkill** | 2633.1 | 0.2 | -38.3 | -1684.7 | NA | -0.2 | 6 | -288.10 | 591.09 | **3.61** | 0.09 | 0.96 | 0.73 |
| Pop + Policy + Prevkill | 355.0 | 0.2 | NA | -1544.7 | NA | -0.5 | 5 | -290.74 | 593.48 | 6.00 | 0.03 | 0.99 | 0.70 |
| Pop + Poplevel + Policy + Prevkill | -582.6 | 0.3 | NA | -1598.7 | -696.9 | -0.4 | 6 | -290.07 | 595.03 | 7.55 | 0.01 | 1.00 | 0.70 |
| Pop + Poplevel + Policy | -694.2 | 0.3 | NA | -2078.7 | -974.6 | NA | 5 | -293.10 | 598.19 | 10.71 | 0.00 | 1.00 | 0.66 |
| Food + Policy + Prevkill | 5744.1 | NA | -58.2 | -2316.1 | NA | 0.3 | 5 | -295.13 | 602.26 | 14.78 | 0.00 | 1.00 | 0.62 |
| Pop + Food + Prevkill | 317.6 | 0.3 | -21.3 | NA | NA | -0.8 | 5 | -299.10 | 610.19 | 22.71 | 0.00 | 1.00 | 0.52 |
| Pop + Poplevel + Food + Prevkill | -167.5 | 0.4 | -24.3 | NA | -463.8 | -0.8 | 6 | -298.92 | 612.73 | 25.25 | 0.00 | 1.00 | 0.51 |
| Pop + Poplevel + Food | 1341.6 | 0.3 | -70.5 | NA | -1108.1 | NA | 5 | -304.62 | 621.24 | 33.76 | 0.00 | 1.00 | 0.35 |

POP = total population; POPLEVEL = bear population >15,000 or <15,000; FOOD = index of wild bear foods during summer and fall; POLICY = whether the MDNR revised nuisance bear policy was in effect (pre- or post-1998); PREVKILL = total human-caused mortality due to hunting and nuisance in previous year.
