# Supplementary material for: Population reduction by hunting helps control human–wildlife conflicts for a species that is a conservation success story
Source: PLoS One. 2020 Aug 11;15(8):e0237274. doi: 10.1371/journal.pone.0237274 (PMC7418986; doi:10.1371/journal.pone.0237274)
Supplement: S2 Table — (DOCX) [file pone.0237274.s003.docx]

**S2 Table.** **Candidate models explaining number of complaints about human–bear conflicts in Minnesota, 1982–2014 (including variable POPF6). Models with ΔAIC(c) ≤ 4 are bolded.**

| Model | Intcept | Coefficients | | | | | k | LogLik | AIC(c) | ΔAIC(c) | w | Cum w | adj.R^2^ |
| --- | --- | --- | --- | --- | --- | --- | --- | --- | --- | --- | --- | --- | --- |
|  |  | pop | food | policy | popF6 | prev  kill |  |  |  |  |  |  |  |
| **PopF6 + Food + policy** | 2812.5 | NA | -45.6 | -1196.4 | 0.7 | NA | 5 | -264.72 | 541.66 | **0.00** | 0.33 | 0.33 | 0.74 |
| **Pop + Food + Policy** | 3265.5 | 0.2 | -50.4 | -1958.5 | NA | NA | 5 | -265.04 | 542.31 | **0.65** | 0.24 | 0.56 | 0.74 |
| **Pop6 + Food + Policy + Prevkill** | 2434.4 | NA | -39.7 | -1017.7 | 0.8 | -0.1 | 6 | -264.55 | 544.32 | **2.67** | 0.09 | 0.65 | 0.74 |
| **Pop + PopF6 + Food + Policy** | 2906.5 | 0.0 | -47.0 | -1410.9 | 0.5 | NA | 6 | -264.65 | 544.53 | **2.87** | 0.08 | 0.73 | 0.74 |
| **Pop + Food + Policy + Prevkill** | 2707.4 | 0.2 | -42.0 | -1789.6 | NA | -0.2 | 6 | -264.67 | 544.58 | **2.92** | 0.08 | 0.80 | 0.74 |
| Pop + PopF6 + Food | 2103.4 | -0.2 | -42.1 | NA | 1.6 | NA | 5 | -266.75 | 545.72 | 4.06 | 0.04 | 0.85 | 0.71 |
| PopF6 + Food + Prevkill | 887.8 | NA | -27.8 | NA | 1.1 | -0.4 | 5 | -266.98 | 546.18 | 4.53 | 0.03 | 0.88 | 0.71 |
| PopF6 + Policy + Prevkill | 15.8 | NA | NA | -708.4 | 0.9 | -0.4 | 5 | -267.02 | 546.26 | 4.60 | 0.03 | 0.91 | 0.71 |
| Pop + Policy + Prevkill | 209.4 | 0.2 | NA | -1625.2 | NA | -0.5 | 5 | -267.44 | 547.11 | 5.45 | 0.02 | 0.94 | 0.70 |
| Pop + PopF6 + Food + Policy + Prevkill | 2457.1 | 0.1 | -40.0 | -1324.7 | 0.5 | -0.2 | 7 | -264.36 | 547.20 | 5.55 | 0.02 | 0.96 | 0.73 |
| Pop + PopF6 + Food + PREVKILL | 1527.9 | -0.1 | -32.5 | NA | 1.5 | -0.2 | 6 | -266.21 | 547.64 | 5.98 | 0.02 | 0.97 | 0.71 |
| Pop + PopF6 + Prevkill | -346.1 | -0.1 | NA | NA | 1.4 | -0.4 | 5 | -267.82 | 547.87 | 6.21 | 0.01 | 0.99 | 0.69 |
| Pop + PopF6 + Policy + Prevkill | 17.4 | 0.1 | NA | -974.3 | 0.7 | -0.4 | 6 | -266.90 | 549.02 | 7.37 | 0.01 | 0.99 | 0.70 |
| Pop + PopF6 + Policy | 101.1 | -0.1 | NA | -1066.9 | 1.0 | NA | 5 | -269.19 | 550.61 | 8.95 | 0.00 | 1.00 | 0.66 |
| Food + Policy + Prevkill | 5951.7 | NA | -63.7 | -2405.1 | NA | 0.4 | 5 | -271.37 | 554.97 | 13.31 | 0.00 | 1.00 | 0.62 |
| Pop + Food + Prevkill | 496.7 | 0.3 | -22.9 | NA | NA | -0.9 | 5 | -275.31 | 562.84 | 21.18 | 0.00 | 1.00 | 0.51 |

POP = total population; POPF6 = population of 6+-year-old females; FOOD = index of wild bear foods during summer and fall; POLICY = whether the MDNR revised nuisance bear policy was in effect (pre- or post-1998); PREVKILL = total human-caused mortality due to hunting and nuisance in previous year.
